# Midkine is a NF-κB-inducible gene that supports prostate cancer cell survival

**DOI:** 10.1186/1755-8794-1-6

**Published:** 2008-02-14

**Authors:** Zongbing You, Ying Dong, Xiangtian Kong, Laurel A Beckett, Regina Gandour-Edwards, Jonathan Melamed

**Affiliations:** 1 Department of Orthopaedic Surgery, University of California, Davis, Sacramento, CA 95817, USA; 2 Department of Pathology, New York University School of Medicine, New York, NY 10016, USA; 3 Department of Public Health Sciences, Division of Biostatistics, University of California, Davis, Sacramento, CA 95817, USA; 4 Department of Medical Pathology and Laboratory Medicine, University of California, Davis, Sacramento, CA 95817, USA; 5 Department of Structural & Cellular Biology and Tulane Cancer Center, Louisiana Cancer Research Consortium, School of Medicine, Tulane University, New Orleans, LA 70112, USA

## Abstract

**Background:**

Midkine is a heparin-binding growth factor that is over-expressed in various human cancers and plays important roles in cell transformation, growth, survival, migration, and angiogenesis. However, little is known about the upstream factors and signaling mechanisms that regulate midkine gene expression.

**Methods:**

Two prostate cancer cell lines LNCaP and PC3 were studied for their expression of midkine. Induction of midkine expression in LNCaP cells by serum, growth factors and cytokines was determined by Western blot analysis and/or real-time quantitative reverse-transcription – polymerase chain reaction (RT-PCR). The cell viability was determined by the trypan blue exclusion assay when the LNCaP cells were treated with tumor necrosis factor alpha (TNFα) and/or recombinant midkine. When the LNCaP cells were treated with recombinant midkine, activation of intracellular signalling pathways was determined by Western blot analysis. Prostate tissue microarray slides containing 129 cases (18 normal prostate tissues, 40 early stage cancers, and 71 late stage cancers) were assessed for midkine expression by immunohistochemical staining.

**Results:**

We identified that fetal bovine serum, some growth factors (epidermal growth factor, androgen, insulin-like growth factor-I, and hepatocyte growth factor) and cytokines (TNFα and interleukin-1beta) induced midkine expression in a human prostate cancer cell line LNCaP cells. TNFα also induced midkine expression in PC3 cells. TNFα was the strongest inducer of midkine expression via nuclear factor-kappa B pathway. Midkine partially inhibited TNFα-induced apoptosis in LNCaP cells. Knockdown of endogenous midkine expression by small interfering RNA enhanced TNFα-induced apoptosis in LNCaP cells. Midkine activated extracellular signal-regulated kinase 1/2 and p38 mitogen-activated protein kinase pathways in LNCaP cells. Furthermore, midkine expression was significantly increased in late stage prostate cancer, which coincides with previously reported high serum levels of TNFα in advanced prostate cancer.

**Conclusion:**

These findings provide the first demonstration that midkine expression is induced by certain growth factors and cytokines, particularly TNFα, which offers new insight into understanding how midkine expression is increased in the late stage prostate cancer.

## Background

Midkine (MDK, or MK) is a 13-kDa heparin-binding growth factor originally identified by screening of retinoic acid-responsive genes [1,2]. MDK plays important roles in the nervous system, inflammation, and cancer [3-5]. MDK has been shown to induce transformation of NIH3T3 cells and to promote cell growth, survival, and migration, as well as angiogenesis [6-10]. Therefore, it is not surprising that MDK has been found to be over-expressed in various human cancers, including esophageal, gastric, colon, pancreatic, hepatocellular, lung, breast, and urinary bladder carcinomas, neuroblastomas, and Wilms' tumors [11,12].

Prostate cancer is the most common malignant disease and the second most common cause of cancer-related death in American men [13]. The patients succumb to androgen-independent cancers that demonstrate alterations in androgen receptor signaling, apoptosis, and neuroendocrine differentiation. Konishi and coworkers first reported that MDK expression was positive in 86.3% of clinical prostate cancer, while normal prostate tissues were negative or showed only weak staining by immunohistochemical staining [14]. They also found that metastatic lesions generally showed higher MDK expression than the corresponding primary tumors. This was supported by a recent report that MDK expression was higher in C4-2 cells (androgen-independent derivative of LNCaP cells, with high tumorigenic and metastatic potential) than in LNCaP cells [15]. However, the biological role of MDK in prostate cancer has not been well addressed.

In this study, we found that fetal bovine serum (FBS) significantly induced MDK expression in LNCaP cells. As the results of searching for the serum factors that induced MDK expression, we identified TNFα as the strongest inducer of MDK expression in LNCaP cells. Further investigation revealed that MDK supported LNCaP cell survival.

## Methods

### Cell culture

Human prostate cancer cell line LNCaP and PC3 cells were from the American Type Culture Collection (Manassas, VA). LNCaP cells were routinely maintained in T-medium (custom formula # 02-0056) with 5% FBS (Invitrogen, Carlsbad, CA). PC3 cells were maintained in Ham's F12K medium with 10% FBS. The cells were cultured in a 37°C, 5% CO_2 _humidified incubator. To avoid any interference from the insulin and triiodothyronine (T3) in the T-medium, the culture medium was switched to serum-free Dulbecco's Modified Eagles Medium (DMEM, Invitrogen, Carlsbad, CA) 16 h after plating the cells for all the experiments in this study. Each experiment was repeated at least twice and only reproducible data were presented in this report.

### Analysis of MDK protein expression by Western blot analysis

500,000 LNCaP cells in one ml 5% FBS T-medium per well were plated in 12-well plates and 16 h later changed into serum-free DMEM with or without growth factors and cytokines. There was no additional treatment during the following 48 h. Control: serum-free DMEM + 1 μl phosphate buffered saline (solvent for growth factors and cytokines) +1 μl ethanol (solvent for DHT and R1881); the concentrations of growth factors and cytokines were: 10 ng/ml recombinant human insulin, 10 ng/ml recombinant human IGF-I, 10 ng/ml recombinant human EGF, 10 ng/ml recombinant human HGF, 10 ng/ml recombinant human bFGF, 20 ng/ml T3, 10 nM DHT, 33.3 μM all-trans-retinoic acid (RA) (Sigma-Aldrich, St. Louis, MO); 10 nM R1881 (synthetic androgen, Perkin-Elmer, Boston, MA); 10 ng/ml TNFα, 10 ng/ml IL-1β, 50 ng/ml IL-6, and 50 ng/ml IL-17 (R&D Systems Inc., Minneapolis, MN). 48 h after treatment, the culture medium was collected and centrifuged at 20,800 × g for 5 min at 4°C. LNCaP cells were also treated with different dosages (1 to 50 ng/ml) of TNFα for 48 h, or 20 ng/ml TNFα for different time periods (8 to 48 h). PC3 cells were also treated with or without 20 ng/ml TNFα in serum-free medium for 48 h. For Western blot analysis, 20 μl of each medium supernatant was subjected to 12% SDS-polyacrylamide gel electrophoresis and transferred to polyvinylidene difluoride membrane by electroblotting. The membranes were blocked with 5% nonfat dry milk in TBST (25 mM Tris-HCl, 125 mM NaCl, 0.1% Tween 20) for 2 hours and probed with rabbit anti-MDK antibodies (PeproTech, Inc., Rocky Hill, NJ) overnight and then horseradish peroxidase-conjungated secondary antibodies for 1 hour. The results were visualized by enhanced chemiluminescence (SuperSignal West Pico Chemiluminescent Substrate, Pierce Biotechnology, Inc., Rockford, IL) according to the manufacturer's instructions. For densitometry, the integrated density values (IDV) of the protein bands were analyzed by FluorChem IS-5500 (Alpha Innotech) and normalized to the control group (arbitrarily assigned a value of 1).

### Analysis of MDK mRNA expression by real-time quantitative RT-PCR

Total RNA was extracted from LNCaP cells not treated or treated with 20 ng/ml TNFα, using RNeasy Mini Kit (QIAGEN, Valencia, CA) with on-membrane DNase I digestion to avoid genomic DNA contamination. cDNA was made from total RNA using Superscript™ First-Strand Synthesis System with oligo dT primers (Invitrogen, Carlsbad, CA). Human MDK and glyceraldehyde-3-phosphate dehydrogenase (GAPDH) primers were obtained from Applied Biosystems (Foster City, CA). Real-time quantitative PCR was done in triplicates with an ABI 7700 Sequence Detector and Sybr-Green reagents (Applied Biosystems) following the recommended protocols [16]. Results were normalized to GAPDH levels using the formula ΔCt (Cycle threshold) = Ct of MDK – Ct of GAPDH. Since LNCaP cells without treatment for 8 h expressed the lowest levels (but detectable, Ct = 18.3 ~ 19.0) of MDK, ΔΔCtwas calculated using the formula ΔΔCt = ΔCt of any group – ΔCt of LNCaP cells without treatment for 8 h. The data were presented as fold change of MDK mRNA compared to LNCaP cells without treatment for 8 h, where fold = 2^ΔΔCt^.

### Inhibition of MDK expression by nuclear factor-κB (NF-κB) inhibitor

LNCaP cells were plated as described previously. They were not treated or treated for 48 h with increasing dosages (from 1 to 20 ng/ml) of TNFα plus constant dosage (18 μM) of a NF-κB inhibitor (Santa Cruz Biotechnology, Inc., Santa Cruz, CA). Alternatively, they were treated for 48 h with constant dosage (20 ng/ml) of TNFα plus increasing dosages (from 2.25 to 18 μM) of NF-κB inhibitor. The medium supernatants were analyzed for MDK protein expression by Western blot analysis as described above.

### Cell viability

**Figure 1:**
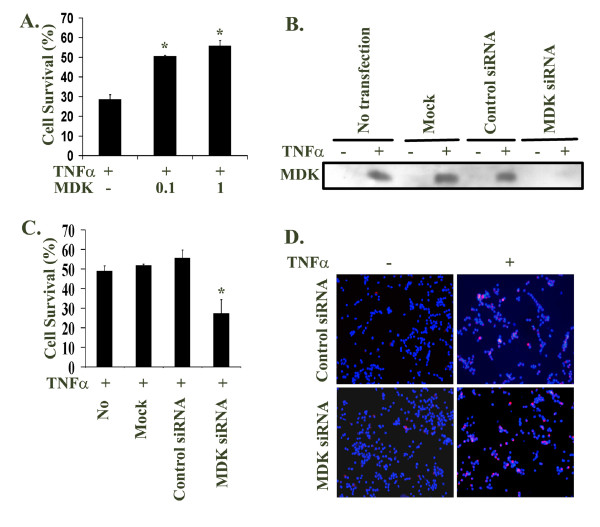
Midkine partially inhibited TNFα-induced apoptosis in LNCaP cells. **A. **LNCaP cells in triplicate groups were not treated (as control) or treated for 4 days with 20 ng/ml TNFα, without or with 0.1 or 1 μg/ml exogenous midkine; the living cell number was counted by the trypan blue exclusion assay; the cell survival was calculated as (the living cell number of treated group ÷ the living cell number of untreated control group); the data were presented as mean ± standard deviation; *P < 0.05 compared to TNFα alone. **B. **LNCaP cells were transfected with the mixtures of midkine specific siRNA/Lipofectamine™ 2000, control-siRNA/Lipofectamine™ 2000, or Lipofectamine™ 2000 only (mock transfection), or no transfection as an additional control; the final concentrations used were 100 nM of siRNA or control-siRNA, and 5 μl/ml of Lipofectamine™ 2000; four h after transfection, the cells were changed into serum-free DMEM without or with 20 ng/ml TNFα; two days later, the medium supernatants were analyzed for midkine expression by Western blot. **C. **LNCaP cells in triplicate groups were treated as described in **B**; the cell survival after 2-days' treatment with 20 ng/ml TNFα was determined by the trypan blue exclusion assay as described in **A**; the data were presented as mean ± standard deviation; *P < 0.05 compared to the other three groups. **D. **LNCaP cells were treated as described in **B**; 16 h after treatment with 20 ng/ml TNFα, 20 μM MR-(DEVD)_2 _were added to the cells and incubated for 1 h, followed by addition of 1 μg/ml Hoechst 33342 for another 15 min; the red fluorescence [emitted by the cleaved MR-(DEVD)_2 _indicating activation of caspase-3] and blue fluorescent nuclei (stained by Hoechst 33342 to illustrate total cell number) were captured by a fluorescent microscope; original magnification: × 100.

20,000 LNCaP cells in one ml 5% FBS T-medium per well in triplicate groups were plated in 12-well plates and 16 h later changed into serum-free DMEM with or without 20 ng/ml TNFα. To test the effect of exogenous MDK on cell survival, 0.1 or 1 μg/ml of recombinant human MDK (PeproTech, Inc., Rocky Hill, NJ) were added 30 min before adding TNFα. Two or four days after treatment, cell viability was determined by the trypan blue exclusion assay, in which cell survival was calculated as (the living cell number of treated group ÷ the living cell number of untreated control group) × 100. Based on our previous experience [17], we chose to determine the cell survival after four-day treatment to better evaluate the protective effects of MDK (Figure 1A), while after two-day treatment to show the combined killing effects of siRNA with TNFα (Figure 1C).

### Knockdown of endogenous MDK expression by small interfering RNA (siRNA)

20,000 LNCaP cells in one ml 5% FBS T-medium per well were plated in 12-well plates and 16 h later transfected with the mixtures of siRNA/Lipofectamine™ 2000 (Invitrogen, Carlsbad, CA), control-siRNA/Lipofectamine™ 2000, or Lipofectamine™ 2000 only (mock transfection), or no transfection as an additional control, according to the manufacturer's protocol [17]. The siRNA was the ON-TARGETplus SMARTpool pre-designed siRNA targeting human MDK (catalog # L-003677-00, Dharmacon, Inc., Lafayette, CO). The control-siRNA was a scrambled Stealth™ siRNA negative control (Invitrogen, Carlsbad, CA). The final concentrations used were 100 nM of siRNA or control-siRNA, and 5 μl/ml of Lipofectamine™ 2000. Four h after transfection, the cells were changed into serum-free DMEM without or with 20 ng/ml TNFα. Two days later, the medium supernatants were analyzed for MDK protein expression by Western blot analysis and the cell viability was determined by the trypan blue exclusion assay as described above. To confirm that the LNCaP cells died through the caspase-3-mediated apoptosis as we observed previously [17], the Caspase 3&7 Magic Red Kit (Immunochemistry Technologies, LLC., Bloomington, MN) was used to detect activated caspase-3. Briefly, a fluorophore (cresyl violet) was coupled to the four amino acid peptides (DEVD, optimal substrate of active caspase-3 & 7) and not able to fluoresce. When active caspase-3 or 7 cleaved off DEVD, cresyl violet emitted red fluorescence. Sixteen h after the siRNA transfection and TNFα treatment, 20 μM MR-(DEVD)_2 _were added to the cells and incubated for 1 h, followed by addition of 1 μg/ml Hoechst 33342 for another 15 min. The red fluorescence (indicating activation of caspase-3) and blue fluorescent nuclei (stained by Hoechst 33342 to illustrate total cell number) were captured by a fluorescent microscope.

### Activation of intracellular signaling pathways by exogenous MDK

Three million LNCaP cells in 10 ml 5% FBS T-medium per dish were plated in 100-mm dishes and 16 h later changed into serum-free DMEM for another 16 h. Then, 100 ng/ml recombinant human MDK was added to the cells for 5 to 480 min. The cells were harvested for protein isolation and Western blot analysis as described previously [16,17]. The antibodies used were: pERK1/2 (pERK, Santa Cruz Biotechnology, Santa Cruz, CA), ERK1/2, P-p38, p38, P-Akt (serine 473), and Akt (Cell Signaling Technology, Beverly, MA). For loading control, the membranes were stripped and probed for GAPDH (antibodies from Chemicon, Temecula, CA). For densitometry, the IDVs of the protein bands were analyzed by FluorChem IS-5500 (Alpha Innotech) and normalized to the control group (arbitrarily assigned a value of 1).

### Immunohistochemical staining

Prostate tissue microarray slides were prepared at the New York University Medical Center and consisted of a total of 132 cases. Each case was represented by two 0.6-mm tissue cores. Among them, 129 cases (18 normal prostate tissues, 40 early stage cancers, and 71 late stage cancers) were analyzed (3 cases lost both tissue cores). The early stage cancer specimens were from radical prostatectomy specimens derived from patients with clinically localized prostate cancer. Half of this group of patients had previously undergone neoadjuvant therapy for 3 months prior to radical prostatectomy. The late stage cancer specimens were from transurethral resection of hormone naïve or resistant prostate cancer that had advanced beyond the stage treatable by radical prostatectomy. Non-tumorous prostate tissues were also derived from the radical prostatectomy specimens. The collection of the specimens was approved by the New York University Institution Review Board.

The prostate tissue microarray slides were stained with 0.6 μg/ml rabbit anti-MDK antibodies using the VECTSTAIN elite ABC Reagent and DAB Substrate Kit (Vector Laboratories, Burlingame, CA) according to the manufacturer's protocol [16,18]. The stained slides were assessed independently by two pathologists (X.K. and J.M.) and a consensus of grading was reached. The evaluation criteria of Konishi et al were adopted, i.e., samples were considered negative (-) if less than 20% of epithelial cells were stained for MDK, weakly positive (+) if 20–50% of epithelial cells were stained, and strongly positive (++) if more than 50% of epithelial cells were stained [14].

### Statistical analysis

The Student's *t*-test was used to analyze the MDK mRNA expression data and the cell survival data. The difference of Gleason scores between the early stage and late stage cancers was analyzed by the Mann-Whitney U test. The immunohistochemical staining data were analyzed by the Kruskal-Wallis test. P < 0.05 was considered statistically significant.

## Results

### Induction of MDK protein expression by growth factors and cytokines

**Figure 2:**
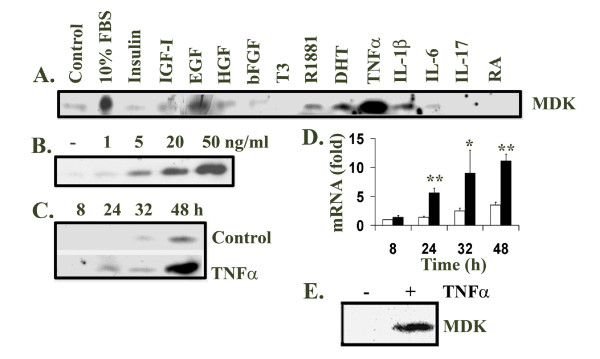
Midkine expression was induced by FBS, growth factors and cytokines. **A**. LNCaP cells were cultured in serum-free DMEM and treated for 48 h with 10% FBS and the indicated agents: 10 ng/ml insulin, 10 ng/ml IGF-I, 10 ng/ml EGF, 10 ng/ml HGF, 10 ng/ml bFGF, 20 ng/ml T3, 10 nM R1881, 10 nM DHT, 10 ng/ml TNFα, 10 ng/ml IL-1β, 50 ng/ml IL-6, 50 ng/ml IL-17, and 33.3 μM RA. **B**. LNCaP cells were treated with different dosages (1 to 50 ng/ml) of TNFα for 48 h. **C**. LNCaP cells were also treated with 20 ng/ml TNFα for different time periods (8 to 48 h). 20 μl of each medium supernatant was subjected to Western blot analysis of midkine expression using rabbit anti-midkine antibodies, horseradish peroxidase-conjungated secondary antibodies and enhanced chemiluminescence reagents. **D**. Total RNA was extracted from LNCaP cells not treated or treated with 20 ng/ml TNFα, using RNeasy Mini Kit; cDNA was made from total RNA using Superscript™ First-Strand Synthesis System with oligo dT primers; real-time quantitative PCR was done in triplicates with Sybr-Green reagents; results were normalized to GAPDH levels as described in Methods; the data (mean ± standard deviation of three independent experiments) were presented as fold change of midkine mRNA compared to the LNCaP cells without treatment for 8 h, where fold = 2^ΔΔCt^; solid bar, TNFα treated; open bar, control; * P < 0.05 and **P < 0.01, compared to the corresponding controls. **E. **PC3 cells were cultured in serum-free medium and treated for 48 h with or without 20 ng/ml TNFα. 20 μl of each medium supernatant was subjected to Western blot analysis of midkine expression.

In this study, we found that FBS induced MDK expression in the human prostate cancer LNCaP cells (Figure 2A). To rule out the possibility that the FBS contained MDK, we loaded the same amount of FBS as in the 10% FBS medium and did not detect any MDK signal (data not shown). This suggested that it was serum factors in the FBS that induced MDK expression in LNCaP cells. Therefore, we tested a panel of thirteen growth factors and cytokines including RA for their effects on MDK induction. Unlike in the teratocarcinoma stem cells, RA did not induce MDK expression in LNCaP cells. Insulin, recombinant human basic fibroblast growth factor (bFGF), triiodothyronine (T3), interleukin-6 (IL-6) and interleukin-17 (IL-17) did not stimulate MDK expression, either (Figure 2A). Recombinant human insulin-like growth factor-I (IGF-I) and hepatocyte growth factor (HGF) slightly induced MDK expression, while epidermal growth factor (EGF), dihydrotestosterone (DHT), R1881 (synthetic androgen), and interleukin-1β (IL-1β) modestly stimulated MDK expression (Figure 2A). However, TNFα induced a dramatic increase in MDK expression and was the strongest inducer among the agents tested (Figure 2A).

### TNFα induced MDK expression through NF-κB pathway

Since TNFα was the strongest inducer of MDK expression in LNCaP cells, we further characterized the induction of MDK expression by TNFα. We found that as low as 1 ng/ml of TNFα slightly induced MDK protein expression and the induction was dose-dependent over a range up to 50 ng/ml (Figure 2B). Furthermore, MDK protein was detected in the cell culture medium after 24 h treatment with 20 ng/ml of TNFα, but not in the untreated control medium at this point (Figure 2C). To determine if the induction of MDK expression was at mRNA level, we did real-time quantitative RT-PCR analysis of MDK mRNA expression. We found that MDK mRNA expression was significantly induced by TNFα after 24 h treatment and continued to increase up to 48 h (Figure 2D). MDK mRNA expression in the untreated cells also gradually and slightly increased over time (Figure 2D), which was consistent with the increase at protein level (Figure 2C). We also found that TNFα induced MDK protein expression in PC3 cells (Figure 2E) and LNCaP sublines C4-2, P151S, R248W and R273H cells [19] (data not shown).

**Figure 3:**
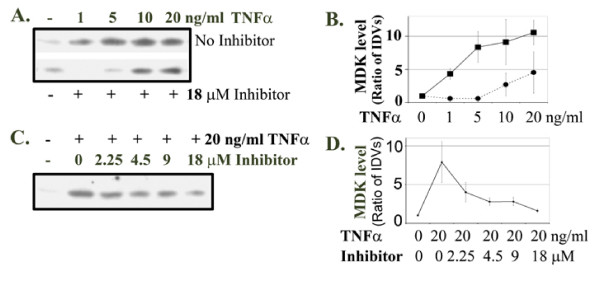
TNFα induced midkine expression through NF-κB pathway. **A. **LNCaP cells were not treated or treated for 48 h with increasing dosages of TNFα, without (top blot) or with 18 μM NF-κB inhibitor (bottom blot); 20 μl of each medium supernatant was subjected to Western blot analysis of midkine expression. **B. **Densitometry of **A**; the untreated control group was arbitrarily assigned a value of 1; the integrated density values (IDVs) of the protein bands from other groups were divided by that of the control group (i.e., ratio of IDVs) to represent the relative MDK levels; solid line, TNFα only; dotted line, TNFα with 18 μM NF-κB inhibitor. Data presented were average ± standard deviations (error bars) of three independent experiments. **C. **LNCaP cells were not treated or treated for 48 h with 20 ng/ml TNFα, and without or with increasing dosages of NF-κB inhibitor; 20 μl of each medium supernatant was subjected to Western blot analysis of midkine expression. **D. **Densitometry of **C **as described in **B**. Data presented were average ± standard deviations (error bars) of three independent experiments.

Since TNFα induces expression of many genes through NF-κB pathway [20], we tested whether induction of MDK expression by TNFα was also mediated by NF-κB pathway. We utilized a synthetic cell-permeable peptide NF-κB inhibitor that inhibits nuclear translocation of NF-κB active complex [21]. We found that 18 μM NF-κB inhibitor completely abolished MDK protein expression induced by 1 ng/ml TNFα, while MDK expression induced by 5 ng/ml TNFα was inhibited to a level slightly below the basal level of the control (Figure 3A and 3B). However, at higher dosages of TNFα, MDK expression was decreased compared to TNFα-treated groups without the NF-κB inhibitor, but its levels were above the basal level (Figure 3A and 3B). In addition, inhibition of MDK induction by the NF-κB inhibitor was in a dose-dependent manner (Figure 3C and 3D).

### MDK partially inhibited TNFα-induced apoptosis in LNCaP cells

Given that MDK was known to prevent apoptosis in other cell types [8,22], we tested whether exogenous MDK could inhibit TNFα-induced apoptosis in LNCaP cells. We found that about 28% of LNCaP cells were alive after being treated with 20 ng/ml TNFα for 4 days, but the cell survival rate was increased to 50% and 56% when 0.1 or 1 μg/ml recombinant human MDK was added (Figure 1A).

In addition, we used MDK-specific siRNA to knockdown the expression of endogenous MDK. As shown in Figure 1B, TNFα induced endogenous MDK expression in the No transfection, Mock transfection, and Control-siRNA transfection groups, but not in the MDK-specific siRNA transfection group. Consequently, TNFα-induced apoptosis in LNCaP cells was enhanced by knocking down endogenous MDK expression. As shown in Figure 1C, the cell survival rates in No transfection, Mock transfection, and Control-siRNA transfection groups were about 50% after being treated by 20 ng/ml TNFα for 2 days, while the cell survival rate in MDK-specific siRNA transfection group was about 25%. To confirm that the LNCaP cells died through caspase-3-mediated apoptosis as we observed previously [17], we used the Caspase 3&7 Magic Red Kit to detect activation of caspase-3. We found that the cells with active caspase-3 were very rare in both Control-siRNA and MDK-specific siRNA transfected groups without TNFα treatment. With TNFα treatment, the Control-siRNA transfected group showed several cells with active caspase-3 (red fluorescence) per microscopic field, while the positive cell number was dramatically increased in the MDK-specific siRNA transfected group (Figure 1D). Since we have determined previously that caspase-7 in LNCaP cells was not activated by TNFα [17], the detected signals were specific for caspase-3.

### MDK activated MAP kinase pathways

**Figure 4:**
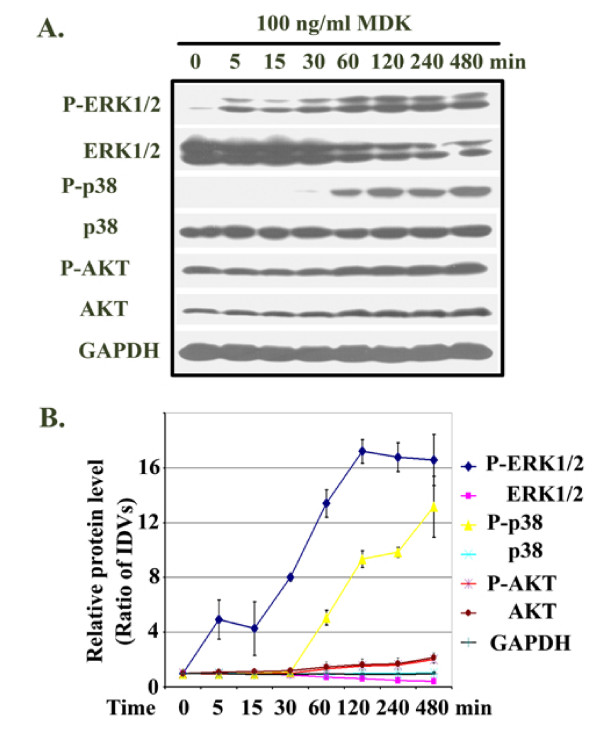
Midkine activated MAP kinase pathways in LNCaP cells. **A. **The serum-starved LNCaP cells were treated with 100 ng/ml recombinant human MDK for 5 to 480 min; the cells were harvested for protein isolation and Western blot analysis of the indicated proteins; for loading control, the membranes were stripped and probed for GAPDH. **B. **Densitometry of **A**; the untreated control group was arbitrarily assigned a value of 1; IDVs of the protein bands from other groups were divided by that of the control group (i.e., ratio of IDVs) to represent the relative individual protein levels over the time course; of note, p38 and GAPDH lines overlapped. Data presented were average ± standard deviations (error bars) of three independent experiments.

As shown in Figure 4A and 4B, MDK induced phosphorylation of ERK1/2 starting 5 min after adding MDK, gradually increasing to a peak at 2 h and sustaining at a lower level thereafter for up to 8 h. It is worth pointing out that the gradual decrease in unphosphorylated ERK1/2 was not caused by unequal loading, as the same samples loaded and probed for both GAPDH and unphosphorylated p38 showed almost equal signals over the time course (Figure 4A and 4B). Phosphorylation of p38 started after 1 h (Figure 4A and 4B). We found high basal levels of phosphorylated Akt and a slight increase in P-Akt and Akt after 1 h (Figure 4A and 4B).

### MDK expression was increased in late stage prostate cancer

**Figure 5:**
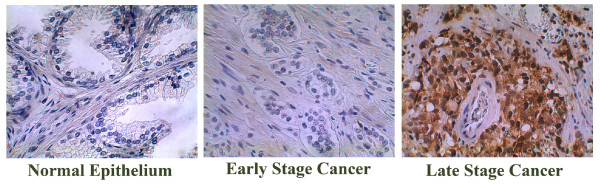
Immunohistochemical staining of prostate tissue microarrays. The early stage cancers were from radical prostatectomy specimens derived from patients with clinically localized prostate cancers; while the late stage cancers were derived from transurethral resection specimens of prostate cancers that had advanced beyond the stage treatable by radical prostatectomy; the normal prostate tissues were from the non-tumorous portions of the radical prostatectomy specimens; the prostate tissue microarray slides were stained with 0.6 μg/ml rabbit anti-midkine antibodies using the VECTSTAIN elite ABC Reagent and DAB Substrate Kit according to the manufacturer's protocol and counter-stained with hematoxylin; representative negative (Normal Epithelium and Early Stage Cancer) and strongly positive (Late Stage Cancer) midkine staining are shown; original magnification: × 400.

By immunohistochemical staining, we found that MDK expression was confined primarily to the cytoplasm of cancer cells and showed a diffuse staining pattern (Figure 5, Late Stage Cancer), which was consistent with the previous report [14]. As shown in Table 1, one out of the 18 normal prostate tissues showed weak staining for MDK, while the rest were negative. Three out of the 40 early stage prostate cancers were weakly positive, while 37 cases were negative. Of the 71 late stage prostate cancers, 6 cases were strongly positive, 24 cases were weakly positive, and 41 cases were negative. Analyzed by Kruskal-Wallis test, MDK expression was significantly higher in the late stage high grade prostate cancers than in the early stage intermediate grade prostate cancers or normal prostate tissues (P < 0.001), while no significant difference was found between the early stage prostate cancers and normal prostate tissues (P > 0.05). It was noted that Gleason scores (indicating tumor differentiation) were significantly higher in the late stage prostate cancers than in the early stage prostate cancers (P < 0.001).

**Table 1:** Midkine expression is increased in the late stage prostate cancers. Normal and prostate cancer tissue microarray slides were immunohistochemically stained for midkine, using rabbit anti-midkine antibodies and ABC elite kit with DAB substrate, and counterstained by hematoxylin. The grading was assigned to each tissue core by a consensus review of two pathologists.

Tissue	No.	Gleason score	P	Midkine stain			P
		**median (range)**		**-**	**+**	**++**	
Normal	18			17	1	0	§
Early stage cancer	40	6 (5 – 7)	*	37	3	0	†
Late stage cancer	71	9 (8–10)		41	24	6	‡

"-" indicates ≤ 20% of epithelial cells were immunopositive;

"+" indicates 20–50% of epithelial cells were immunopositive;

"++" indicates ≥ 50% of epithelial cells were immunopositive.

* P < 0.001, Gleason scores, early stage cancer v.s. late stage cancer.

§ P > 0.05, normal v.s. early stage cancer.

† P < 0.001, early stage cancer v.s. late stage cancer.

‡ P < 0.001, normal v.s. late stage cancer.

## Discussion

MDK has been shown to play an important role in development, cell survival, inflammation and tumorigenesis. In spite of its restricted expression in adult kidney, MDK expression is increased in disease conditions such as rheumatoid arthritis and cancer [5,12,23]. Intensive studies have been performed on how and what MDK can do in the various physiological and pathological processes. However, the upstream factors and signaling mechanisms that regulate MDK expression have not been identified. MDK was first identified as a retinoic acid-responsive gene in the teratocarcinoma stem cells [1]. Recently, MDK was found to be upregulated by hypoxia but downregulated by cortisol [24,25]. Rebamipide, a mucoprotective drug used for the treatment of gastric ulcers, has been shown to upregulate MDK expression in rat gastric mucosal cells [26]. In this study, we found that fetal bovine serum stimulated MDK expression in a prostate cancer cell line LNCaP. Furthermore, we found that EGF, DHT, R1881, and IL-1β modestly stimulated MDK expression, while IGF-I and HGF slightly induced MDK expression. TNFα was identified as the strongest inducer of MDK expression in LNCaP cells, while insulin, bFGF, T3, IL-6, and IL-17 did not induce MDK expression in LNCaP cells. This, to our best knowledge, was the first time to identify these growth factors and cytokines as MDK inducers. It was reasonable to speculate that induction of MDK expression by fetal bovine serum might be a result of combined effects of low levels of TNFα, EGF, DHT, IL-1β, IGF-I, and HGF existing in the serum. Surprisingly, RA did not induce MDK expression in LNCaP cells, indicating a cell-specific effect of RA.

We found that TNFα induced MDK expression in a dose-dependent and time-dependent manner at protein levels. At mRNA levels, TNFα-induction of MDK expression was significant after 8 h, indicating that MDK may belong to the late-response genes [27]. More importantly, induction of MDK expression by TNFα was through NF-κB pathway, as NF-κB inhibitor could dose-dependently inhibit TNFα-induced MDK expression. Uehara and co-workers have previously predicted a putative NF-κB-binding site in the 5' non-coding region of MDK gene [28]. In this study, we provided the first direct evidence that MDK was a NF-κB-inducible gene.

MDK has previously been shown to prevent apoptosis in other cell types [8,22]. In this study, we found that exogenously added recombinant human MDK partially inhibited TNFα-induced apoptosis in LNCaP cells. Knocking down the endogenous MDK expression by siRNA enhanced TNFα-induced apoptosis through activation of caspase-3 in LNCaP cells. These data suggested that MDK supported LNCaP cell survival, although its strength was not sufficient to prevent the eventual cell death in this in vitro cell model. Our study showed that MDK also activated ERK1/2 pathway as early as within 5 minutes of treatment in LNCaP cells. Activation of PI3k/Akt pathway by MDK was less obvious in LNCaP cells, due to the constitutively high basal level of activity [29]. Furthermore, we found p38 pathway was dramatically activated by MDK after one hour treatment, while c-jun NH2 terminal kinase pathway was not activated (data not shown). Activation of the ERK1/2 pathway has been reported to primarily results in cell survival, while the role of p38 pathway is less clear, although recent studies indicate a role in cell survival in certain cell types [30-32]. The ERK1/2 pathway has been shown to induce BAD phosphorylation at Ser^112 ^and thereby protect LNCaP cells from apoptosis [33,34]. In previous studies, MDK has been shown to inhibit apoptosis through activation of ERK1/2 and PI3k/Akt pathways in neurons and cardiomyocytes [8,22,35]. It remains to be tested if the same mechanism is true in prostate cancer cells.

MDK has been found to be over-expressed in various human cancers, including esophageal, gastric, colon, pancreatic, hepatocellular, lung, breast, and urinary bladder carcinomas, as well as neuroblastomas and Wilms' tumors [11,12]. MDK expression in prostate cancer tissues was reported by one group [14]. In this study, we found that the normal prostate tissues and early stage intermediate grade prostate cancers are negative or only weakly positive for MDK expression, while MDK expression is significantly increased in the late stage prostate cancers with higher Gleason scores. Konishi and co-workers have previously reported that there appears to be a significant positive correlation of MDK immunoreactivity and higher Gleason scores [14]. However, it was noteworthy that only 42% of the late stage cancers and 8% of the early stage cancers were positive for MDK staining in the current study, while 79% of the biopsied cancers (including 89% of cancers with Gleason's pattern ≥ 4 and 68% of cancers with Gleason's pattern ≤ 3) were positive in the previous study [14]. This disparity may be due to using different antibodies and staining protocols, or different sampling. Nevertheless, Michalaki and co-workers reported that the serum levels of TNFα in patients with locally advanced/metastatic prostate cancers were about 4 to 6 times of those in normal men, while the serum levels of TNFα in patients with localized prostate cancers were only 1.3 times of those in normal men [36]. Therefore, it is possible that increased MDK expression the in late stage prostate cancers may be induced by increased systemic and/or local TNFα and other factors as identified in this study.

What remains not clear is that TNFα eventually kills LNCaP cells in vitro, but the in vivo tumors continue to grow in the presence of high TNFα. One possibility is that the TNFα dosage used in our study is significantly higher than the in vivo concentration of TNFα. It has been reported that the normal physiological serum levels of TNFα vary from 1 to 13 pg/ml [36-39]. Under pathological conditions, serum TNFα levels can be as high as 53 pg/ml in advanced stage of cancer [39], or even 368 pg/ml in rheumatoid arthritis patients [37]. The local tissue TNFα levels may be higher than the serum levels as serum TNFα has a very short half-life [40,41]. It may also be possible that TNFα induces not only MDK expression, but also expression of other anti-apoptotic genes in vivo. MDK may promote angiogenesis in vivo and therefore provide further support for tumor growth [42]. Combination of MDK specific siRNA and chemotherapy may be a promising strategy for the late stage prostate cancers as shown by a recent study [43].

## Conclusion

This study provides the first demonstration that midkine expression is induced by certain growth factors and cytokines, particularly TNFα, which offers new insight into understanding how midkine expression is increased in the late stage prostate cancers. Given the anti-apoptotic role of MDK in prostate cancer cells, our findings further support the new strategy to target MDK using specific siRNA as shown by a recent study [43].

## Abbreviations

bFGF, basic fibroblast growth factor; Ct, cycle threshold; DMEM, Dulbecco's Modified Eagles Medium; DHT, dihydrotestosterone; EGF, epidermal growth factor; ERK, extracellular signal-regulated kinase; FBS, fetal bovine serum; GAPDH, glyceraldehyde-3-phosphate dehydrogenase; HGF, hepatocyte growth factor; IDV, integrated density values; IGF-I, insulin-like growth factor-I; IL-1β, interleukin-1 beta; IL-6, interleukin-6; IL-17, interleukin-17; IL-17RL, interleukin-17 receptor-like; MAP, mitogen-activated protein; MDK or MK, midkine; NF-κB, nuclear factor-kappa B; PI3K, phosphatidyl-inositol 3-kinase; RA, retinoic acid; RT-PCR, reverse transcription-polymerase chain reaction; siRNA, small interfering RNA; T3, triiodothyronine; TNF, tumor necrosis factor.

## Competing interests

The author(s) declare that they have no competing interests.

## Authors' contributions

ZY conceived and designed the study, performed the in vitro experiments, and drafted the manuscript. YD and RGE did immunohistochemical staining. XK and JM provided the prostate tissue microarray slides and evaluated the stained slides. LAB contributed to the design and statistical analysis. All authors were involved in preparing the manuscript.

## Pre-publication history

The pre-publication history for this paper can be accessed here:


